# C-arm computed tomography and volume perfusion computed tomography (VPCT)-based assessment of blood volume changes in hepatocellular carcinoma in prediction of midterm tumor response to transarterial chemoembolization: a single center retrospective trial

**DOI:** 10.1186/s40644-016-0088-y

**Published:** 2016-09-21

**Authors:** Roland Syha, Sergios Gatidis, Gerd Grözinger, Ulrich Grosse, Michael Maurer, Lars Zender, Marius Horger, Konstantin Nikolaou, Dominik Ketelsen

**Affiliations:** 1 Department of Radiology, Diagnostic and Interventional Radiology, Eberhard-Karls-University, Hoppe-Seyler-Str. 3, 72076 Tübingen, Germany; 2 Department of Internal Medicine I, Division of Translational Gastrointestinal Oncology, University of Tuebingen, Tuebingen, Germany

**Keywords:** Hepatocellular carcinoma, Transarterial chemoembolization, Drug eluting beads, Volume perfusion computed tomography, C-arm computed tomography, Parenchymal blood volume, Treatment monitoring

## Abstract

**Background:**

This study aims to evaluate immediate changes in perfusion parameters in hepatocellular carcinoma (HCC) to transarterial chemoembolization (TACE) in C-arm computed tomography (CT) and volume perfusion CT (VPCT) and prediction of midterm tumor response.

**Methods:**

Twenty-five patients (median age 66, range 61 to 75 years) with 62 HCC lesions undergoing TACE received immediate pre- and post-interventional assessment by C-arm CT and VPCT. Cross-sectional imaging was analyzed at baseline and approximately 12 weeks after TACE according to modified RECIST criteria. Outcome was defined as objective response (OR, > 30 % reduction of viable tumor) or non-OR. Perfusion parameters were evaluated in C-arm CT [parenchymal blood volume (PBV)] and VPCT [blood volume (BV) and blood flow (BF)]. Ratios of perfusion parameters before and after TACE within the tumor and the non-affected liver parenchyma were calculated.

**Results:**

Correlation between tumor PBV and BV revealed a moderate correlation (rho = 0.45, *p* = 0.005). In non-affected liver parenchyma, a significant decrease in PBV was seen, compared to a significant increase in BF and BV. Perfusion ratios in HCC lesions were significantly (*p* < 0.05) increased in OR group compared to non-OR patients in C-arm CT and VPCT: PBV ratio (0.95 (0.06) to 0.67 (0.38), BV ratio 0.63 (0.34) to 0.15 (0.6), and BF ratio 0.6 (0.32) to 0.22 (0.51). Logistic regression including PBV and BF allowed prediction of OR (sensitivity 88 %/specificity of 83 %).

**Conclusions:**

Perfusion parameters acquired by C-arm CT and VPCT cannot simply be substituted by each other, but show similar capability in prediction of midterm tumor response.

## Background

In intermediate staged hepatocellular carcinoma (HCC), transarterial chemoembolization (TACE) is recommended in liver-only disease [1, 2]. Besides conventional TACE, TACE with drug eluting beads (DEB-TACE) is increasingly being used due to a reported lower liver toxicity and better tolerability [3].

For both methods, a super-selective angiographic approach is favorable and clearly recommended [4–6]. For detection of tumor feeding vessels and smaller HCC lesions, which are partly difficult to identify in conventional angiography, C-arm CT during the intervention with direct, selective, intra-arterial contrast media application is increasingly used [7–9]. The use of C-arm CT results in substantial gain of additional and essential information for treatment planning [7, 10]. In up to one-third of the cases, information gained during C-arm CT acquisition may substantially impact treatment strategy [9, 10]. Besides acquisition of contrast enhanced C-arm CT, nowadays, dual phase C-arm CT acquisitions are possible [11]. The acquisition of more than one contrast phase allows more complex image post-processing, including calculation of absolute quantitative perfusion parameters. The parameter “parenchyma blood volume” (PBV) has recently been introduced. Derived from a non-enhanced and a contrast enhanced C-arm CT acquisition in the steady state of liver perfusion it offers substantial information on the arterial blood volume of liver parenchyma and on that of hyper-vascularized liver tumors [10, 12–15]. Perfusion parameters are of increasing interest in the diagnosis and evaluation of hepatocellular carcinoma and have been evaluated in volume perfusion computed tomography (VPCT) in previous studies [16–18]. VPCT enables measurement of tissue attenuation in a pre-selected volume at different time points. For calculation of different perfusion parameters such as blood volume (BV) and blood flow (BF) ROIs in the feeding artery and the corresponding tissue are placed. From these ROIs, perfusion parameters can be derived using different mathematical models. Most frequently used models are single-compartment analysis (maximum slope) and two-compartment models (deconvolution as well as Patlak analysis) [19, 20]. PBV uses only two different time points (non-enhanced and contrast-enhanced run) assuming that blood volume refers to the amount of blood which is present at a given moment and can therefore be assumed to be constant during the time of acquisition [10].

Both methods show potential for prediction of treatment effects of conventional as well as DEB-TACE, e.g., showing areas of residual perfusion immediately after TACE, these being lesions with a known unfavorable outcome [12–14, 16, 18]. A detailed comparison of both methods concerning mid-term treatment response in DEB-TACE in HCC has not been performed to date.

Therefore, the aim of this retrospective study was to evaluate the clinical value of assessing immediate changes in perfusion parameters in terms of perfusion ratios assessed by C-arm CT and VPCT, acquired before and after DEB-TACE, for prediction of mid-term tumor HCC response. Furthermore, comparability and correlation between both imaging methods for detection of blood volume (BV) was performed using different reconstruction models of CT perfusion data.

## Methods

### Study population

**Fig. 1:**
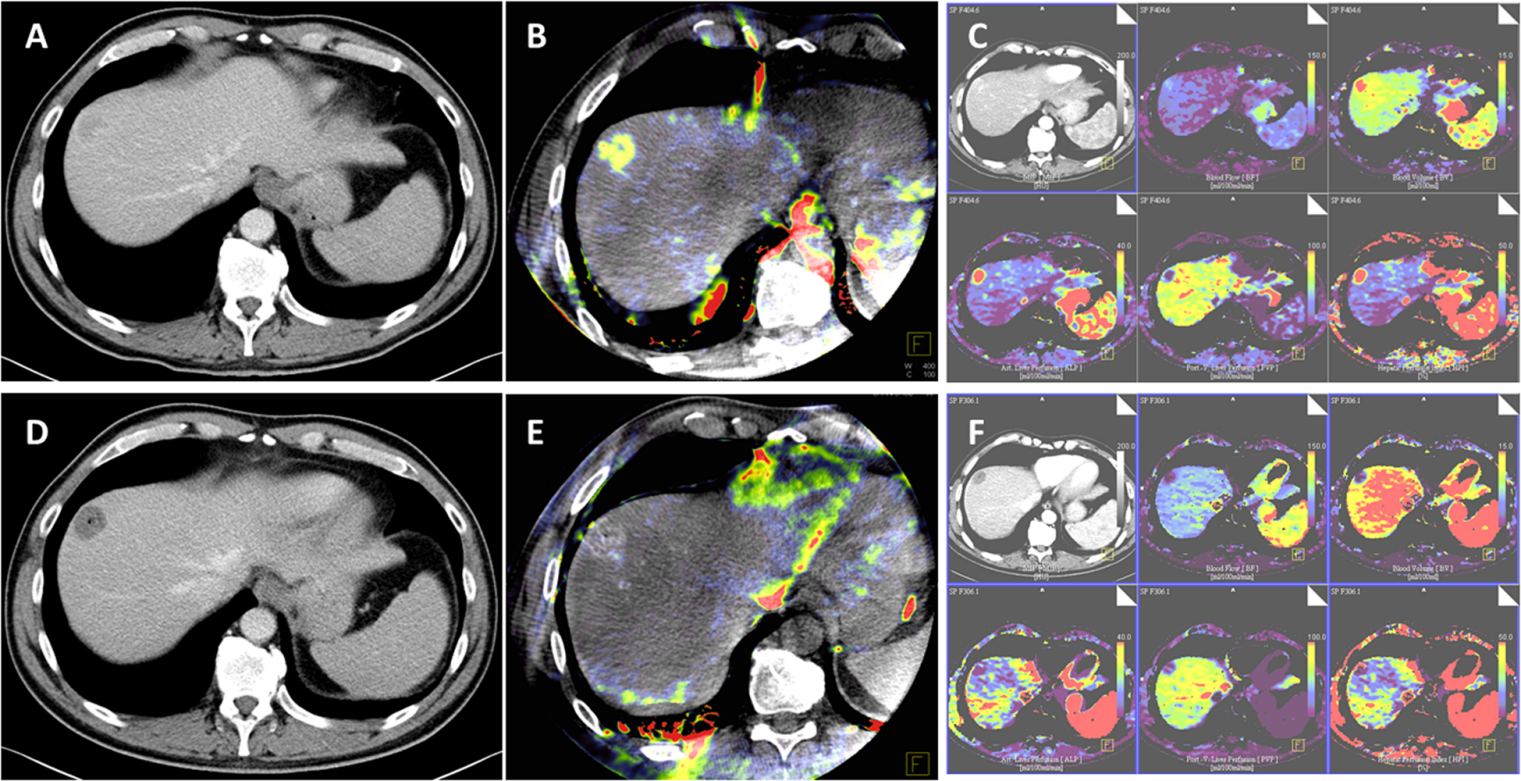
The Figure presents an exemplary case of a patient undergoing DEB-TACE. Pre-interventional CT (**a**) and VPCT (**c**) show a hypervascularized tumor in Segment 8 of the liver with increased blood volume, blood flow and arterial liver perfusion. Corresponding, **b** shows demarcation of a HCC lesion with increased intra-tumoral parenchymal blood volume (PBV) in the C-arm CT acquisition. **d**-**f** present the corresponding imaging techniques after successful DEB-TACE. **e** shows complete reduction of PBV in the PBV maps and typical staining of contrast media within the lesion in the overlaid non-enhanced C-arm CT acquisition

From January 2014 to June 2015, a total of 25 patients (median age, 66; range, 61 to 75 years) received DEB-TACE in combination with pre- and post-interventional C-arm CT and VPCT at our institution (Fig. 1). All of them were included in this retrospective study. Main inclusion criterion for performing TACE in our institution is tumor stage BCLC B (intermediate stage HCC). Furthermore, TACE was performed in tumor stage BCLC A (early stage HCC) if curative treatment such as resection or radiofrequency ablation was initially not possible or as a bridging treatment before orthotropic liver transplantation [1]. Exclusion criteria were adopted from the guidelines of international interventional radiology societies including general exclusion criteria [4]. General exclusion criteria were elevated bilirubin (>2 mg/dl), a lactate dehydrogenase level (425 mg/dL), an aspartate aminotransferase level (100 IU/l), poor liver function (Child C) and poor patient’s performance status (ECOG > 2). Furthermore, extrahepatic spread and portal vein thrombosis were also exclusion criteria as well as a poor metabolic status (Serum creatinine 2 mg/dL, Platelet count 50,000/mm^3^, Prothrombin activity 50 %).

Underlying disease was hepatitis B or hepatitis C virus infection (hepatitis B, *n* = 2; hepatitis C, *n* = 4; both, *n* = 3), alcoholic liver disease (*n* = 5), non-alcoholic steatohepatitis (*n* = 3), hemochromatosis (*n* = 1), mixed liver disease (*n* = 1), or cryptogenic (*n* = 6). Detailed patient information is provided in Table 1.

**Table 1:** Baseline characteristics

Data		Value
Age (years)		
	Mean (SD)	67.2 (±9.41)
Sex		
	Male	24
	Female	1
Child-Pugh Classification		
	Child A	23
	Child B	2
MELD score		
Pre TACE	Mean (SD)	7.4 (±1.15)
Post TACE	Mean (SD)	7.53 (±1.7)
No. of nodules		
	1	8
	2	8
	3	7
	4	2
Lesion size (mm)	mRECIST	
	Mean (SD)	24.9 (±13.3)
	< 3 cm	36
	≥ 3 cm	17
Previous number of TACEs		
	0	10
	1	6
	2	6
	>2	3

### Baseline and follow-up imaging

All patients received CT imaging at baseline, prior to DEB-TACE on the same 128-row CT scanner (Somatom Definition AS+, Siemens Healthcare, Forchheim, Germany) including a three-phasic protocol with non-enhanced images (40 mAs, 100 kV, SL = 5.0 mm, collimation128 mm × 0.6 mm, tube rotation time 0.5 s, pitch 0.6), arterial, and portal-venous phase. Approximately 12 weeks after successful DEB-TACE, follow-up cross-sectional imaging (CSI) was performed. All cross-sectional images were assessed according to the modified response evaluation criteria in solid tumors (mRECIST) for HCC: [1] progressive disease (PD, an increase of at least 20 % in diameter of viable tumor); stable disease (SD, any cases that do not qualify for either partial response or progressive disease); partial response (PR, at least a 30 % decrease in diameter of viable tumor); complete response (CR, disappearance of any intratumoral arterial enhancement) [21]. Furthermore, all CR and PR were subsumed as objective response (OR). All patients (patient based) and HCC lesions (lesion based) were evaluated according to mRECIST criteria for response evaluation of HCC. All data analyses were performed by the same board certified interventional radiologist.

### Volume perfusion computed tomography

VPCTs were gained during standard baseline CT imaging (see above), and during the same hospital visit, immediately after DEB-TACE was performed. The non-enhanced CT scan of the standard CT acquisition (see above) was used for planning of the scan range of 6.9–9.7 cm z-axis coverage of VPCT scan. Afterwards, VPCT was performed covering if possible the whole liver or at least the affected parts of the liver (80 kV, 100–120 mAs for patients </> 70 kg, respectively, collimation64 × 0.6 mm with z-flying focal spot, 26 repeated-CT-scans, total scan time 40 s) according to previous studies in VPCT for assessment of DEB-TACE [18].

All perfusion analyses were performed at the same workstation (Syngo MMWP,VE36A, Siemens Healthcare) using the software package Syngo Volume Perfusion CT body (Siemens Healthcare).

After automated motion correction and noise reduction by an algorithm with non-rigid deformable registration for anatomic alignment, regions of interest (ROIs) were put in the lumen of the abdominal aorta, the portal vein, and the spleen parenchyma to separate arterial and portal venous phase according to previous studies investigating VPCT in TACE [18]. Volumes of interest were put in tumor areas with maximum perfusion before and after performed DEB-TACE. MIP (maximum intensity projections) reconstruction of VPCT scans were used as anatomical reference for volume of interest (VOI) placement. Furthermore, three VOIs were put in the non-affected liver parenchyma to calculate the average liver perfusion. The following quantitative perfusion parameters were evaluated: [1] blood flow (BF) in ml/100 ml/min, [2] blood volume (BV) in ml/100 ml, [3] arterial liver perfusion (ALP) in ml/100 ml/min, and [4] portal venous perfusion (PVP) in ml/100 ml/min. The hepatic perfusion index (HPI) in percentage was derived from ALP and PVP.

Secondly, perfusion analysis was performed using an arterial only input function. For this purpose, a ROI was put in the abdominal aorta. BF in ml/100 ml/min and BV in ml/100 ml were calculated using different estimation models: [1] maximum slope model (MS), [2] Patlak analysis (P), [3] deconvolution (D)). For this purpose, VOIs were set in the MIP reconstruction of perfusion scans in areas of maximum tumor perfusion. Additionally, three reference VOIs were put in the non-affected liver parenchyma, to calculate the average liver perfusion.

### Angiography and DEB-TACE

All interventions were performed in the same robotic angiography suite (Artis Zeego Q, Siemens Healthcare, Forchheim, Germany). The arterial system was accessed by puncture of the right common femoral artery, after introduction of a 4 F sheath (Terumo, Leuven, Belgium) over a 0.035 in. guidewire (Terumo, Leuven, Belgium). In cases of unclear origin of the hepatic arteries a straight diagnostic catheter was introduced and aortography was performed. The celiac trunk was accessed with a cobra (C2) or sidewinder (SIM1) configurated catheter (Cordis, Fremont, California, U.S.) and celiacography was performed. After selective catheterization of the common hepatic artery, overview angiography was performed for assessment of the number and extent of tumor blushes and tumor feeding vessels. A 2.7 F microcatheter (Progreat, Terumo) was introduced for super selective catheterization of the tumor feeders. Epirubicin loaded microparticles (100–300 μm, DC-Beads, BTG, Langweid/Augsburg, Germany) were cautiously applied under fluoroscopy control until near stasis was reached, according to the guidelines of DEB-TACE [4]. Finally, completion angiography from the common hepatic artery was performed.

### C-arm computed tomography and parenchymal blood volume

Before and after delivery of DEB, a C-arm CT acquisition was performed, with the diagnostic catheter placed in the common hepatic artery, according to previous studies [10, 13]. C-arm CT consisted of a mask non-enhanced run, a return run, and a fill run (4 s) with a gap of 2 s between the different runs (total acquisition time = 16 s). After mask run, a total of 30 ml diluted contrast media [7,5 ml Ultravist 370 (Bayer Schering, Leverkusen, Germany) and 22,5 ml saline solution] was administered via automated power injection (Accutron-HP-D, Medtron, Saarbrücken, Germany). The start of contrast media application was manually triggered after the fill run. The flow rate was set to 3 ml/s. This guaranteed a contrast enhanced acquisition in a steady-state phase of liver perfusion according to previous studies dealing with PBV of the liver [10, 12, 15].

All post-processing was performed at the same workstation with an implemented commercially available software package (syngo XWP, Siemens Healthcare). Motion correction between non-enhanced and contrast enhanced run was performed using a non-rigid registration algorithm. Automated histogram analysis of the vessel tree was used to create the steady state arterial input function followed by a final scaling of the datasets to account for the arterial input value. Afterwards noise reduction was achieved using a smoothing filter [12, 15].

VOIs were put in that part of the HCC lesion with maximum PBV in ml/100 ml before and after DEB-TACE. Furthermore three VOIs were set in the non-affected liver parenchyma for calculation of average liver PBV.

### Data analysis

All assessed PBV values were compared to different calculation algorithms of BV in VPCT. All detected HCC lesion in CSI were classified in small lesions (<3 cm) and large lesions (≥3 cm) according to a previous study on PBV in HCC [15] for correlation between different BV assessment and PBV in dependency of lesion diameter.

For all assessed PBV values in HCC lesions, ratios between pre- and post-interventional C-arm CT acquisition were performed according to the formula: Ratio (PBV) = (PBV pre-PBV post)/PBV pre.

Concordantly, ratios between pre- and post-interventional VPCT values were calculated according to the formula: Ratio (value) = (VPCT (value) pre-VPCT (value) post)/VPCT (value) pre for BF, BV, ALP, and HPI as well as according to the formula: Ratio (PVP) = (PVP post-PVP pre)/PVP post.

### Statistics

All statistics were performed using the statistical software package JMP (SAS, Cary, NC). Arithmetic mean and standard deviation or median and interquartile ranges were given for descriptive statistics. The Wilcoxon signed-rank test was used to test for significance. The Mc Nemar test was used for comparison of dichotomous data. A *p*-value below 0.05 was accepted as significant difference. Correlation between different blood volume assessments was performed using Spearman’s rho. To predict the influences on response rate (OR) a logistic regression was performed. Sensitivity and specificity of the model was calculated using Receiver operating curves (ROC) analyzing. In the primary model all relevant arterial perfusion parameters were integrated including ratios of ALP, BF, BV, and PBV. Subsequently non-significant parameters were excluded from logistic regression until a model with only significant influences (*p* < 0.05) was calculated.

## Results

### Overall results

Median time between baseline imaging and DEB-TACE was 2 days (range, 1 to 7 days). Time between DEB-TACE and post-TACE VPCT was 3 days (range, 2 to 8 days). Median interval between baseline and follow-up cross-sectional imaging was 80 days (range, 51 to 100 days). Imaging follow-up was available in 22/25 patients and consisted of CT (*n* = 19) or MRI (*n* = 3). In 3/25 patients, no imaging follow-up was available: of these three patients, 1/3 patient received OLT before imaging follow-up, 1/3 patients showed decompensated liver function (Child C) and was treated by best supportive care, and 1/3 patients was lost in follow-up. Patient based treatment response according to mRECIST was CR in 6/22 patients, PR in 8/22, SD 4/22, and PD in 4/22. This resulted in a significant reduction of baseline sum diameter from 51.1 mm (±21.6 mm) to 28.6 (±26.5 mm) in follow-up CSI.

Lesion-based treatment response at mid-term (*n* = 46) according to mRECIST was CR in 21/46 (46 %), PR in 7/46 (15 %), SD in 14/46 (30 %), and PD in 4/46 (9 %). This resulted in a significant reduction of maximum lesion diameter from 24.9 mm (±13.3 mm) to 13.9 mm (±17.9 mm) (*p* > 0.0001).

Combining all three methods (CSI, VPCT, and C-arm CT), a total of 62 lesions were detected. In the baseline assessment, a total of 53/62 HCC lesions were detected in cross-sectional images. In VPCT images, a total of 58/62 HCC suspected lesions were detected and in PBV maps, a total 59/62 HCC suspected lesions were detected. There was no significant difference between PBV maps, VPCT, and CSI concerning lesion detectability.

Regarding perfusion parameters before and after DEB-TACE, a significant change was seen in HCC lesions, with a decrease of PBV, BV, BF, and ALP. In non-affected liver parenchyma, a significant decrease in PBV was seen, compared to a significant increase in BF, BV, and ALP after DEB-TACE. Detailed information on perfusion parameters including arithmetic mean, standard deviations, and *p*-values is summed up in Table 2.

**Table 2:** Perfusion parameters gained by VPCT and C-arm CT before and after TACE in HCC lesions and non-affected liver parenchyma

Parameter	Number	Pre TACE	Post TACE	*p*-value
HCC lesions				
PBV (ml/100 ml)	59	17.16 (±5.9)	2.7 (±4.2)	<0.0001
BF (ml/100 ml/min)	58	57.8 (±19.22)	26.58 (±19.87)	<0.0001
BV (ml/100 ml)	58	13.75 (±3.89)	7.06 (±5.07)	<0.0001
ALP (ml/100 ml/min)	58	47.40 (±15.02)	12.02 (±17.85)	<0.0001
HPI (%)	58	87.64 (±23.3)	24.72 (±32.35)	<0.0001
Non affected liver parenchyma				
PBV (ml/100 ml)	25	3.16 (±1.67)	2.48 (±1.71)	0.0059
BF (ml/100 ml/min)	25	24.76 (±15.59)	34.13 (±16.66)	0.0005
BV (ml/100 ml)	25	7.23 (±3.67)	8.92 (±3.75)	0.0015
ALP (ml/100 ml/min)	25	10.23 (±7.08)	12.72 (±10.59)	0.1108
PVP (ml/100 ml/min)	25	62.68 (±19.31)	60.14 (±21.12)	0.6099
HPI (%)	25	16.22 (±10.03)	19.2 (±15.61)	0.4078

The *p*-value indicates the level of significance. Arithmetic mean and standard deviation are given

*Abbreviations: PBV* parenchymal blood volume, *BF* blood flow, *BV* blood volume, *ALP* arterial liver perfusion, *PVP* portal venous perfusion, *HPI* hepatic perfusion index, *n* number of cases

### Tumor PBV in correlation to different BVs assessed by VPCT

Overall correlation between tumor PBV and different BV revealed a moderate correlation for PBV and BV (MS) (rho = 0.45, *p* = 0.005) as well as for PBV and BV (P) (rho = 0.3, *p* = 0.0237). For PBV and BV (D), only a non-significant weak correlation was seen (rho = 0.24, *p* = 0.08). Regarding dependency of measured perfusion parameters on lesion extent, a moderate correlation between PBV and BV (MS) (rho = 0.44, *p* = 0.0127) was found in smaller lesions (<3 cm). Only a weak, non-significant correlation was seen for PBV and BV (P) (rho = 0.27, *p* = 0.1481) and PBV and BV (D) (rho = 0.26, *p* = 0.15). A moderate correlation was seen for PBV and BV (MS) (rho = 0.6, *p* = 0.0112), for PBV and BV (P) (rho = 0.54, *p* = 0.0255), as well as for PBV and BV (D) (rho = 0.5, *p* = 0.041) in lesions ≥ 3 cm. Detailed information on different BV methods and PBV is given in Table 3.

**Table 3:** Comparison of different blood volumes acquired by VPCT to PBV gained during C-arm CT before TACE

Parameter	Number	Value (ml/100 ml)
PBV	59	17.14 (±5.9)
BV (MS)	58	13.36 (±4.55)
BV (P)	58	14.22 (±17.69)
BV (D)	58	16.42 (±7.04)

Arithmetic mean and standard deviation are given

*Abbreviations: PBV* parenchymal blood volume, *BV (MS)* blood volume maximum slope, *BV(P)* blood volume Patlak analysis, *BV(D)* blood volume deconvolution

### VPCT perfusion parameters and C-arm CT based PBV in prediction of midterm tumor response

Ratios of PBV and perfusion parameters (BF, BV, ALP, and HPI) show increased values in OR group compared to patients with SD or PD. Arithmetic mean and standard deviations as well as levels of significance were summed up in Table 4. In non-affected liver parenchyma, ratios of PVP and HPI showed significant higher ratios of HPI and PVP for OR group (-0.08 ± 0.93 and 0.1 ± 0.34) compared to patients with SD or PD [HPI ratio: -0.62 ± 1.33 (*p* = 0.0491); PVP ratio: -0.16 ± 0.42 (*p* = 0.0383)]

**Table 4:** Comparison of lesions with OR to no OR

Parameter	OR	SD, PD	*p*-value
n	26	18	n. a.
Baseline mRECIST (mm)	23.16 (±10.32)	28.89 (±16.1)	0.4749
PBV ratio	0.95 (±0.06)	0.67 (±0.38)	0.0303
ALP ratio	0.84 (±0.3)	0.43 (±0.66)	0.0163
HPI ratio	0.77 (±0.44)	0.47 (±0.58)	0.0521
BV ratio	0.63 (±0.34)	0.15 (±0.6)	0.0023
BF ratio	0.6 (±0.32)	0.22 (±0.51)	0.008

Ratios of perfusion parameters gained by C-arm CT and VPCT are given. The *p*-value indicates the level of significance. Arithmetic mean and standard deviation are given

*Abbreviations: PBV* parenchymal blood volume, *BF* blood flow, *BV* blood volume, *ALP* arterial liver perfusion, *HPI* hepatic perfusion index, *n* number of cases, *n.a.* not applicable

Logistic regression was performed for ratios of all relevant arterial perfusion parameters (BV, BF, ALP, PBV) and baseline diameter according mRECIST for all treated HCC lesions with follow-up in CSI (*n* = 46). Regarding the regression model (1 = OR; 0 = SD or PD), best differentiation between groups was reached for ratios of BF (MS) and PBV with a sensitivity of 88 % and a specificity of 83 % in ROC analysis.

## Discussion

This retrospective study focuses on the usefulness of early perfusion measurements by VPCT and C-arm CT for prediction of midterm tumor response to TACE in HCC. Perfusion analysis for evaluation of treatment response has been evaluated in previous studies concerning PBV (C-arm CT), VPCT, dynamic contrast enhanced MRI, and contrast enhanced ultrasound. A decrease in arterial liver perfusion parameters seems to be associated with a favorable outcome after treatment [18, 22, 23].

Regarding perfusion parameters acquired by VPCT before and after TACE, our results yielded comparable results to previous studies evaluating TACE in HCC [16, 18]. The same applies when comparing results of PBV measurement in HCC lesions and healthy liver parenchyma of our study to previous published data [10, 13]. Comparing perfusion parameters acquired by VPCT and those assessed by C-arm CT (PBV) revealed some interesting additional information provided by C-arm CT, indicating an adaptation in tumor and healthy liver parenchyma to treatment. While PBV decreases in healthy and affected liver parenchyma as an immediate response to treatment, in healthy liver parenchyma BV and BF show a significant increase in VPCT performed in a median time interval of 3 days. Contrarily, tumor BF and BV remains below the initial value, which can be interpreted as a reaction to treatment. This highlights potential differences between both methods with have to be taken into consideration when comparing the assessment of treatment response in PBV maps or in VPCT maps.

Previous studies comparing PBV and VPCT-based BV-values showed a very high correlation between both methods in the pre-interventional setting (correlation coefficient above 0.9) [12, 15]. These results are only partially supported by our study. For different assessments of VPCT-BV, our study revealed only a moderate correlation between BV and PBV in the pre-interventional setting. The best correlation was seen for PBV and BV (MS). The differences to previously published studies might be for several reasons. Firstly, some studies evaluated a smaller sample size (20 lesions and 14 lesions) [12, 15]. Secondly, Peynircioğlu et al. investigated a quite inhomogenous study population, including HCC and metastatic lesions [12]. It is well known that hypovascular metastases and HCC lesion show different perfusion patterns [24]. Thirdly, the lesion size of assessed HCC lesions was systemically higher in the study of Zhuang et al. In our institution, large HCC lesions (>5 cm) are not routinely treated by TACE, according to the HAP 2 criteria [25]. Therefore, we additionally investigated the influence of lesion size on comparability of BV and PBV. Correlation coefficient was higher regarding HCC lesions ≥ 3 cm when compared to lesion below 3 cm for all assessed BV (MS, P, and D). We conclude that comparison of BV and PBV has to be regarded with care in case of small HCC lesions and therefore cannot be simply substituted by each other in the pre-interventional setting. When comparing results of PBV maps to VPCT parameters, the maximum slope model for calculation of BV and BF should preferably be applied.

Concerning the assessment of treatment response, previous studies showed that the pre-interventional amount of blood volume and blood flow does not seem to be an adequate surrogate parameter for mid-term tumor response after TACE. Responder and Non-responder showed similar perfusion parameters before intervention. Contrarily, the reduction of arterial perfusion parameters after performed TACE is postulated to have potential as a surrogate parameter of mid-term tumor response [16, 18]. A similar finding has recently been published concerning the assessment of PBV maps. Areas of residual increased PBV seem to be associated with an unfavorable outcome in mid-term tumor response [13]. We therefore concluded that absolute perfusion values before and after treatment might not be ideal predictors for treatment response, but ratios of perfusion parameters before and after DEB-TACE should be more appropriate for the assessment of treatment response. This has already been underlined by a previous perfusion study dealing with mid-term treatment response in lymphoma [26].

Our study shows that patients with an OR approximately 3 months after DEB-TACE showed significantly increased arterial perfusion and blood volume ratios in HCC lesions immediately after TACE, as compared to patients with SD or PD. As PBV maps acquired by C-arm CT and perfusion parameters in VPCT showed different adaptations after treatment, we decided to run a regression model, including PBV and BF, to deal with advantages of both methods. Of course, this model has to be regarded with care due to the limited sample size of our retrospective study, but clearly underlines the potential of both methods in prediction of outcome after DEB-TACE in patients with HCC (sensitivity of 88 % and specificity of 83 %).

These findings could indicate a change in re-treatment schedule after TACE, if residual perfusion is present after performed DEB-TACE. Possible options might be a shortened re-treatment interval or a change of therapeutic conception (e.g., radioembolization) in patients with inadequate response in immediate perfusion assessment.

### Limitations

Mean weakness of the logistic regression model is the small sample size. So results have to be regarded with care. Nevertheless tendency towards a good a priori differentiation of mid-term tumor response in DEB-TACE using immediately before or after intervention acquired perfusion parameters is underlined by this study. These findings have to be verified in a large cohort to define clear cut-off values for further treatment planning. A histologic correlation was not possible in this retrospective study population.

## Conclusion

This study underlines the usefulness of perfusion parameters in the immediate evaluation of treatment response after DEB-TACE. Ratios of perfusion parameters before and after intervention seem to be most appropriate. PBV acquired by C-arm CT and perfusion parameters in VPCT cannot simply be substituted by each other, but nevertheless show similarly capability in determination of treatment effects. This capability increases when both methods are used complementarily. HCC lesions as well as healthy liver parenchyma show an adaptation to treatment with an initial decrease in tumor PBV immediate after intervention and a compensatory increase of blood volume within a week after treatment in VPCT.

## Abbreviations

ALP: Arterial liver perfusion
BF: Blood flow
BV: Blood volume
C2: Cobra
CR: Complete response
CSI: Cross-sectional imaging
CT: Computed tomography
D: Deconvulation
DEB: Drug eluting beads
HCC: Hepatocellular carcinoma
HPI: Hepatic perfusion index
MIP: Maximum intensity projections
mRECIST: Modified response evaluation criteria in solid tumors
MRI: Magnetic resonance imaging
MS: Maximum slope model
OR: Objective response
P: Patlak analysis
PBV: Parenchymal blood volume
PD: Progressive disease
PR: Partial response
PVP: Portal vein perfusion
ROC: Receiver operating curve
ROI: Region of interest
SD: Stable disease
SIM1: Sidewinder
TACE: Transarterial chemoembolization
VOI: Volume of interest
VPCT: Volume perfusion CT
